# Use of Photodynamic Therapy for Treatment of Actinic Keratoses in Organ Transplant Recipients

**DOI:** 10.1155/2013/349526

**Published:** 2012-12-24

**Authors:** Christina Wlodek, Faisal R. Ali, John T. Lear

**Affiliations:** ^1^St Thomas' Hospital, Westminster Bridge Road, London SE1 7EH, UK; ^2^The Dermatology Centre, Manchester Academic Health Science Centre, University of Manchester, Salford Royal NHS Foundation Trust, Manchester M6 8HD, UK

## Abstract

Solid organ transplant recipients are predisposed to actinic keratoses (AK) and nonmelanoma skin cancers, owing to the lifelong immunosuppression required. Today, increasing numbers of organ transplants are being performed and organ transplant recipients (OTRs) are surviving much longer. Photodynamic therapy (PDT) is proving a highly effective treatment modality for AK amongst this susceptible group of patients. Following an overview of the pathogenesis of AK amongst OTRs, the authors review current safety and efficacy data and how this relates to the role of PDT for the treatment of AK in OTRs.

## 1. Introduction

Actinic keratoses (AK), also known as solar keratoses, are the commonest premalignant dermatological pathology, clinically manifest as hyperkeratotic papules and plaques with an erythematous base and superficial scale with histology demonstrating intraepidermal proliferation of atypical keratinocytes [1]. In addition to cumulative ultraviolet (UV) radiation exposure, particularly UVB, which is the main risk factor for development of AK and nonmelanoma skin cancer (NMSC) in the general population, susceptibility in organ transplant recipients (OTRs) is significantly increased by long-term exposure to immunosuppressive medications.

## 2. Burden of NMSC in Transplant Patients

Precancerous lesions, including AK and Bowen's disease, are more common in transplant recipients affecting up to 40% within 5 years of transplantation [2]. Furthermore, organ transplant recipients have shown between a 65 and 250-fold increased risk of developing squamous cell carcinoma (SCC) and a 10-fold increased risk of developing basal cell carcinoma (BCC) [3–5].

Skin malignancy is the leading cause of mortality in this group of immunosuppressed patients. The average time for developing SCC is less than 9 years after transplant [6, 7]. These lesions tend to be more aggressive with an increased propensity for recurrence and metastasis despite treatment [5, 6, 8, 9]. Moreover, these patients are prone to developing multiple NMSCs with a 5-year cumulative risk of approximately 70% [10, 11].

Several changes in transplantation trends are responsible for the increasing burden of NMSC observed in these patients. Firstly, the number of transplant procedures continues to increase, partly attributable to the ageing population. With better surgical techniques and more effective immunosuppressive regimes and medical management, recipients are surviving longer [12] and patients are being transplanted at an older age.

## 3. Role of Immunosuppressive Medications in AK and NMSC Pathogenesis

Immunosuppressive drugs can inhibit proinflammatory cytokines, affect the p53 tumour suppressor gene, and increase susceptibility to human papilloma virus infection [13–15].

Immunosuppressive medications increase susceptibility to premalignant and malignant skin lesions in a variety of ways. Ciclosporin, a commonly used calcineurin inhibitor (CNI), interferes with repair of damaged DNA and UVB-induced keratinocyte apoptosis [16, 17]. It has also been demonstrated to stimulate synthesis of tumour growth factor beta, which increases tumour invasiveness and metastatic potential [18, 19]. Other CNIs, including tacrolimus, generate a variety of cytokines promoting angiogenesis and facilitating tumour growth and metastasis [20]. A metabolite of azathioprine, 6-thioguanine, can accumulate in keratinocyte DNA rendering it more sensitive to UVA radiation, with subsequent formation of oxidative DNA photoproducts, increasing the risk of mutagenesis [21].

These findings have led to the development of an alternative class of immunosuppressants—the inhibitors of the mammalian target of rapamycin (mTOR) including everolimus and sirolimus. These drugs exhibit antitumour activity via their inhibition of angiogenesis as well as cell cycle progression and growth. Rival-Tringali et al. demonstrated that switching from a CNI to sirolimus reduced vascularization and thickness of posttransplant human cutaneous SCC *in vivo* [22]. Following a recent meeting of pan-European dermatology experts, transplant physicians are being recommended to have recipients switched to these more protective, antineoplastic medications, particularly in patients with documented SCC [13].

Evidence from a variety of organ transplants has revealed that the greater the dose and the longer the duration of immunosuppressive medication OTRs receive, the greater their propensity to develop NMSCs [23, 24].

## 4. Management of AK and NMSC in OTRs

As advised by the National Institute of Clinical Excellence in the UK, organ transplant recipients with AK should be reviewed regularly in a secondary care skin surveillance clinic. The main challenge lies in finely balancing the need to prevent transplant rejection whilst minimising the risk of immunosuppression and its promalignant complications. One of the mainstays of achieving this balance is through active patient education. This includes sun avoidance and regular use of sunscreens, evidenced by a prospective trial that showed daily sunscreen application for 2 years in OTRs resulted in a reduced number of AK and SCC compared to those who did not follow this regimen [25]. Explaining the need for skin vigilance coupled with regular dermatology review is vital. In view of this, it has been suggested each patient has an evaluation of dermatological risk factors (both intrinsic and extrinsic) after transplantation such that a predictive risk for the development of NMSC can be established early after transplant [26].

There are several options available for the active treatment of AK. It is important to distinguish between lesion-directed and field-directed therapies, as in practice most patients will receive both. The most widely used management choice, for small isolated lesions, in immunocompetent individuals is cryotherapy with liquid nitrogen [27]. Cryotherapy is also useful in OTRs to treat solitary AK.

The idea of field change was first described in 1953, and more recently this concept has been demonstrated at molecular level. Patches of genetically altered stem cell clones develop into individual fields that eventually mature into contiguous pastures of precancerous cells [28]. This mandates field therapy rather than targeting individual lesions. Further support for this management concept comes from data showing 82% of SCCs arising within, in close proximity to, or in a region contiguous with AK. Furthermore, it is acknowledged that the risk of surrounding skin to develop SCC is reduced if AK lesions are treated [29]. Indeed areas with multiple AK are largely accountable for NMSC-related morbidity and mortality and hence successful treatment should to be field directed [30].

The use of topical therapies, whilst effective, is often limited by the protracted course of treatment required and local side-effects including skin irritation, dryness, erythema, and exfoliation, which are often poorly tolerated by patients. One of the most widely recognised topical agents is 5-fluorouracil (5-FU), a chemotherapeutic agent, which works by inhibiting thymidylate synthetase and consequently disrupting DNA synthesis. In otherwise healthy individuals, when used twice daily for 3 weeks, 5-FU is associated with a reduction of lesional area by 70% [31]. However, a trial comparing its use with PDT in OTRs found it to be significantly less effective [32].

Imiquimod (5%) cream is an immune response modifier that acts via stimulation of a Toll-like receptor culminating in malignant cell apoptosis and a local Th1-based immune response. Randomised controlled trials have demonstrated its efficacy both clinically and histologically over a 16-week treatment course [33, 34]. Complete clinical clearance was achieved in 47% of patients and partial in 64%. Furthermore this efficacy has been echoed in OTRs even though it lacks official approval for this patient group [35]. Nevertheless it should be used with caution in this population given its potential to increase systemic interferon levels and hence theoretically the risk of graft rejection [13]. It is more expensive than 5-FU with a similar side effect profile, including severe erythema as well as scabbing and crusting (30%) with some ulceration (10%) [36].

Diclofenac gel, a topical nonsteroidal anti-inflammatory agent, is another treatment option for AK. Although not specifically licensed for OTRs, it does have a safe evidence base, with a complete clearance rate of 41% and a 53% reduction in the number of individual lesions after four weeks of treatment [37].

## 5. Photodynamic Therapy

PDT combines the use of a photosensitising drug with targeted phototherapy to act on rapidly dividing atypical keratinocytes with treatment rates in immunocompetent individuals approaching 90% [27, 38, 39]. PDT is particularly useful when lesions are numerous and located in areas of poor wound healing [36, 40]. In a randomized intraindividual study, Morton et al. compared PDT with double freeze-thaw cryotherapy in otherwise healthy individuals, repeating treatments at three months if required. After 24 weeks both groups had similarly high response rates but cosmetic outcome and satisfaction were significantly higher in the PDT group [41]. PDT with either 5-aminolevulinic acid (ALA) or methylaminolevulinic acid (MAL) is a well-recognised treatment modality for AK [42] as well as superficial and nodular BCC in OTRs [43].

The success of PDT relies upon adequate penetration of the relevant wavelengths of light, hence sufficient production of protoporphyrin IX, together with optimal cellular uptake of photosensitizer. The former can be negatively affected if lesions are particularly hyperkeratotic, as is often the case with AKs in OTRs [44, 45]. In the case of stubborn NMSCs, it has proved useful to aggressively debulk tumours prior to PDT with a variety of methods including curettage [46–48], keratolytics [49], fractional laser techniques [50], and laser ablation [51]. Hence, clinical assessment of AK and appropriate preconditioning of lesions are paramount for optimal treatment with PDT.

Dragieva et al. were the first to compare the use of PDT for AK and Bowen's disease in a group of immunocompetent patients compared to those on immunosuppressants. In the first instance, 4 weeks after intervention, the complete clearance rate was comparable between the two groups (94% and 86%). However, in the longer term (48 weeks after treatment) the results were much poorer with complete clearance being achieved in 48% of the immunocompromised population compared to 72% in the control group [32]. Echoing the findings in immunocompetent patients, the final therapeutic PDT outcome is better on the face and scalp compared to the hands [52, 53]. Analysis of the trials thus far suggests at least two PDT sessions should be performed in field cancerisation areas at baseline (1-2 weeks apart), followed by further sessions several times per year. This regimen appears to provide adequate clearance of AK for up to 3 months in OTRs [13, 54].

In addition to the markedly hyperkeratotic nature of many lesions seen in OTRs, another contributory factor to the inferior efficacy of PDT upon a given lesion in the OTR population is the impaired ability of immunosuppressed OTRs to mount an adequate immune response. The mode of action of PDT relies in part on its induction of a local immune reaction that in turn eliminates mutagenic cells. Unsurprisingly therefore, this response will be hampered in immunosuppressed individuals [55]. This was confirmed in the case of BCC in OTRs, whereby the density of the perilesional inflammatory infiltrate was significantly reduced compared to immunocompetent cases [56].

One study comparing MAL-PDT to 5-FU demonstrated a complete clearance rate at 4 weeks of 89% with MAL-PDT compared to just 11% with 5-FU. Despite pain being a considerable side effect, PDT offered the best cosmetic outcome and was the preferred treatment option by patients [42].

In addition to short-term aesthetic benefit, evidence supports the role of PDT in reducing carcinogenesis. Bagazgoitia et al. demonstrated its action at molecular level through decreased expression of p53, which is a marker of early skin cancer [54].

Cyclical ALA-PDT has also been shown to reduce the incidence of new SCC in OTRs, which when used for two years, has been shown to lead to a mean reduction in new SCC and Bowen's disease of 95% compared to baseline [57]. Further support for the use of PDT in prevention of NMSC came from a Danish group who showed that one session of MAL PDT in renal transplant patients significantly increased the mean time to occurrence of a new NMSC lesion to 9.6 months (versus 6.8 months in controls) [58], findings that have recently been validated in a separate population, confirming that regular field PDT has the potential to prevent AK in the OTR population [59].

Although the number of reported studies so far is small, PDT in OTRs appears to be more effective for BCC than AK with remission rates of 75% after 12 weeks, compared to 48% in AK [60], and a low recurrence rate of under 6% after 22 months of followup [43, 61].

## 6. Adverse Events of PDT

An analysis of all the reported adverse events, from studies involving over 200 OTRs who underwent PDT, revealed no damage to the graft [13]. The risk of further immunosuppression from PDT, particularly in OTRs, is however undefined due to limited investigations to date. However, given the knowledge that PDT not only recruits proinflammatory factors but also local immunosuppressive interleukins and tumour necrosis factor alpha [62, 63], there is a real possibility that further local immunosuppression may impair the response to infection. In keeping with this, recently it has been suggested that there is a suppressed local Mantoux response in healthy volunteers who have received MAL- and ALA-PDT [64]; however, these results have not yet been independently replicated. Furthermore, there is a documented case of confined herpes simplex reactivation in a patient who received PDT to the forehand for AK [65]. As a means to limit this phenomenon, it has been proposed that by reducing the rate of irradiation, whilst maintaining the same light dose, immunosuppression can be prevented [66]. To date, there is no compelling evidence that PDT causes long-term deleterious immunosuppression in patients with AK or other NMSC.

Owing to pain being the most common and limiting side effect of PDT, a number of studies have looked into analgesic options. Although there is no direct evidence, OTRs seem to be more affected than other individuals, particularly with lesions on the head and scalp requiring field therapy. This is likely to reflect size, number, and induration of their lesions compared to immunocompetent patients [67]. It seems that nerve blocks provide relatively uncomplicated yet effective pain relief for patients requiring extensive treatment, particularly when involving sensitive areas on the face and scalp, with significantly reduced pain visual analogue scores reported within a field to which a nerve block was applied compared to the untreated side [68, 69]. Other options reported include transcutaneous electrical nerve stimulation (TENS) [70] and simple cold air fans [71] and cold water sprays [72]. Curiously, topical lidocaine and similar products appear to be no better than placebo [73].

## 7. Alternative Emerging Treatment Modalities

Ingenol mebutate, derived from the plant *Euphorbia peplus,* was licensed for use in AK by the U.S. Food and Drug Administration in January 2012. This novel treatment has a dual mode of action via both cellular necrosis and neutrophil-mediated, antibody-dependent cellular cytotoxicity [74, 75]. Phase III trial data suggested that just two days of field-directed application of 0.05% ingenol mebutate on the trunk and extremities, or three days of field-directed application of 0.015% ingenol mebutate, are significantly more efficacious than vehicle control with respect to complete and partial clearance of lesions [76]. The new product also appears to be well-tolerated and is likely to exhibit high adherence rates given that only a short duration of application is required [77].

Another plant extract showing promise in the treatment of AK is betulinic acid. Its chemical structure is that of pentacyclic triterpenoid, which is extracted from birch bark. In addition to its antimicrobial and antiviral properties, it has more recently been discovered to induce apoptosis and hence shown promise as an antineoplastic agent [78]. A randomised comparative Phase IIa study with 45 patients, each with less than 10 facial and scalp AKs, showed a complete clearance rate of 64% after twice daily application of betulin-based oleogel for a total of 3 months, compared to 79% with cryotherapy and 71% with a combination of the two treatments [79].

An immune response modifier, resiquimod, has similar molecular effects to imiquimod and is another emergent topical treatment of AK. It also acts as an agonist at Toll-like receptors 7 and 8, however, is more potent than its counterpart with the induction of additional chemokines and interleukins resulting in the activation of myeloid and plasmacytoid dendritic cells [80]. A phase II dosing study of resiquimod gel in 132 patients used once daily application, three times per week, for 4 weeks to a contiguous 25 cm^2^ area baring four to eight lesions. The escalating gel concentrations showed a range of complete clearance rates from 40% to 70%. Unsurprisingly, tolerance was greatest with 0.01% and 0.03% concentrations [81, 82].

## 8. Conclusion

NMSCs, including AKs, are a common problem in OTRs who are maintained on lifelong immunosuppressive agents. Management begins with prevention through patient education of sun protective measures and reduction of dose of immunosuppressants with the possible use of less harmful immunosuppressive drugs such as mTOR inhibitors. 

PDT is a safe, well-tolerated treatment option for NMSCs and remains an effective treatment modality for OTRs. However, it should be recalled that PDT appears to be less effective in the longer term for individual lesions in immunosuppressed patients compared to the healthy population, and therefore OTRs need earlier and numerous treatment at regular intervals to obtain the best response and possibly prevent development of new AK and NMSC.
